# Supplementary material for: Alteration of ribosome function upon 5-fluorouracil treatment favors cancer cell drug-tolerance
Source: Nat Commun. 2022 Jan 10;13:173. doi: 10.1038/s41467-021-27847-8 (PMC8748862; doi:10.1038/s41467-021-27847-8)
Supplement: Supplementary file 1 — Supplementary Information [file 41467_2021_27847_MOESM1_ESM.pdf]

Alteration of ribosome function upon 5-fluorouracil treatment favors cancer cell drug-tolerance

Therizols, G. et al.

SUPPLEMENTARY MATERIAL

SUPPLEMENTARY METHODS

Electron microscopy. Cells were grown in a 6-well plate for 24 h and fixed with 2% glutaraldehyde (Sigma) in 0.1 M Sodium Cacodylate buffer at room temperature for 30 min. After washing three times in 0.2 M Sodium Cacodylate buffer, cell cultures were post-fixed with 2% aqueous Osmium Tetroxide (Sigma) and dehydrated in a graded series of ethanol at room temperature and embedded in Epon (Polysciences). After polymerization, ultrathin sections were collected on 300 mesh grids coated with Formvar (SPI supply) and stained with aqueous 1% uranyl acetate (SPI supply) and citrate (Leïca Ultrostainer) before observation on a Philips CM 120 transmission electron microscope at an acceleration voltage of 80 kV.

Northern blot analysis. Northern blot was performed as described in [1]. The probes were obtained by oligonucleotide synthesis (Eurogenetec) and are described in Extended data Table 1. 50 pmoles of each oligonucleotide probe was labelled in the presence of 50 pmoles of [γ-^{32}P] ATP (Perkin Elmer) and T4 polynucleotide kinase (NEB) for 30 min at 37°C. 3 µg of nuclear RNAs were resolved on a 1% denaturing agarose gel and blotted onto a Hybond-N+ membrane (GE Healthcare). Signal detection was performed using a PhosphorImager (FLA 9500, GE Healthcare). Total 28S and 18S rRNA were visualised by fluorescence imaging following ethidium bromide staining and were used as loading controls. Radioactivity was measured for each band and normalised against 18S and 28S rRNA signals stained with ethidium bromide as loading references. Quantification was performed using ImageJ (https://imagej.net) [2].

High throughput automated live cell counting by HCS (High Content Screening). Cell counting by HCS was performed on a Perkin Elmer Operetta-CLS system, with a 20x NA 0.4 objective. Image acquisition was performed using Harmony software (Perkin Elmer). Analysis was performed on 25

fields per well. Hoechst 33342 fluorescence was measured using a 355-385 nm filter for excitation and a 430-500 nm filter for emission. Image analysis and nucleus counting were performed with the Columbus software (Perkin-Elmer).

Supplementary references

1. Erales, J. *et al.* Evidence for rRNA 2'-O-methylation plasticity: Control of intrinsic translational capabilities of human ribosomes. *Proc. Natl. Acad. Sci.* **8**, 201707674 (2017).
2. Schneider, C. A., Rasband, W. S. & Eliceiri, K. W. NIH Image to ImageJ: 25 years of image analysis. *Nat. Methods* **9**, 671–5 (2012).
3. Ahmed, D. *et al.* Epigenetic and genetic features of 24 colon cancer cell lines. *Oncogenesis* **2**, (2013).

Supplementary data Table 1: CRC cell lines characteristics

	TP53	K-Ras	BRAF	MSI status	CIN	Reference
**HCT116**	WT	G13D	WT	MSI	-	Ahmed, D., et al. 2013 [3]
**HT29**	R273H	WT	V600E	MSS	+	Ahmed, D., et al. 2013 [3]
**SW480**	R273H	G12V	WT	MSS	+	Ahmed, D., et al. 2013 [3]
**ISRECO-1**	Y163H	G12D	WT	MSS	+	Ahmed, D., et al. 2013 [3]

Supplementary data Table 2: Sequence of oligonucleotides used in this study

Target	Application	Primer	Sequence
**Actin**	RT-qPCR	F	ATGATATCGCCGCGCTCG
		R	CGCTCGGTGAGGATCTTCA
**GAPDH**	RT-qPCR	F	AGCCACATCGCTCAGACAC
		R	GCCCAATACGACCAAATCC
**IGF-1R**	RT-qPCR	F	AAAAACCTTCGCCTCATCC
		R	TGGTTGTCGAGGACGTAGAAA
**Pre-rRNA ETS1**	Northern blot		CGCTAGAGAAGGCTTTTCTC
**Pre-rRNA ITS1**	Northern blot		CCTCTTCGGGGGACGCGCGCGTGGCCCCGA
**Pre-rRNA ITS2**	Northern blot		GCGCGACGGCGGACGACACCGCGGCGTC

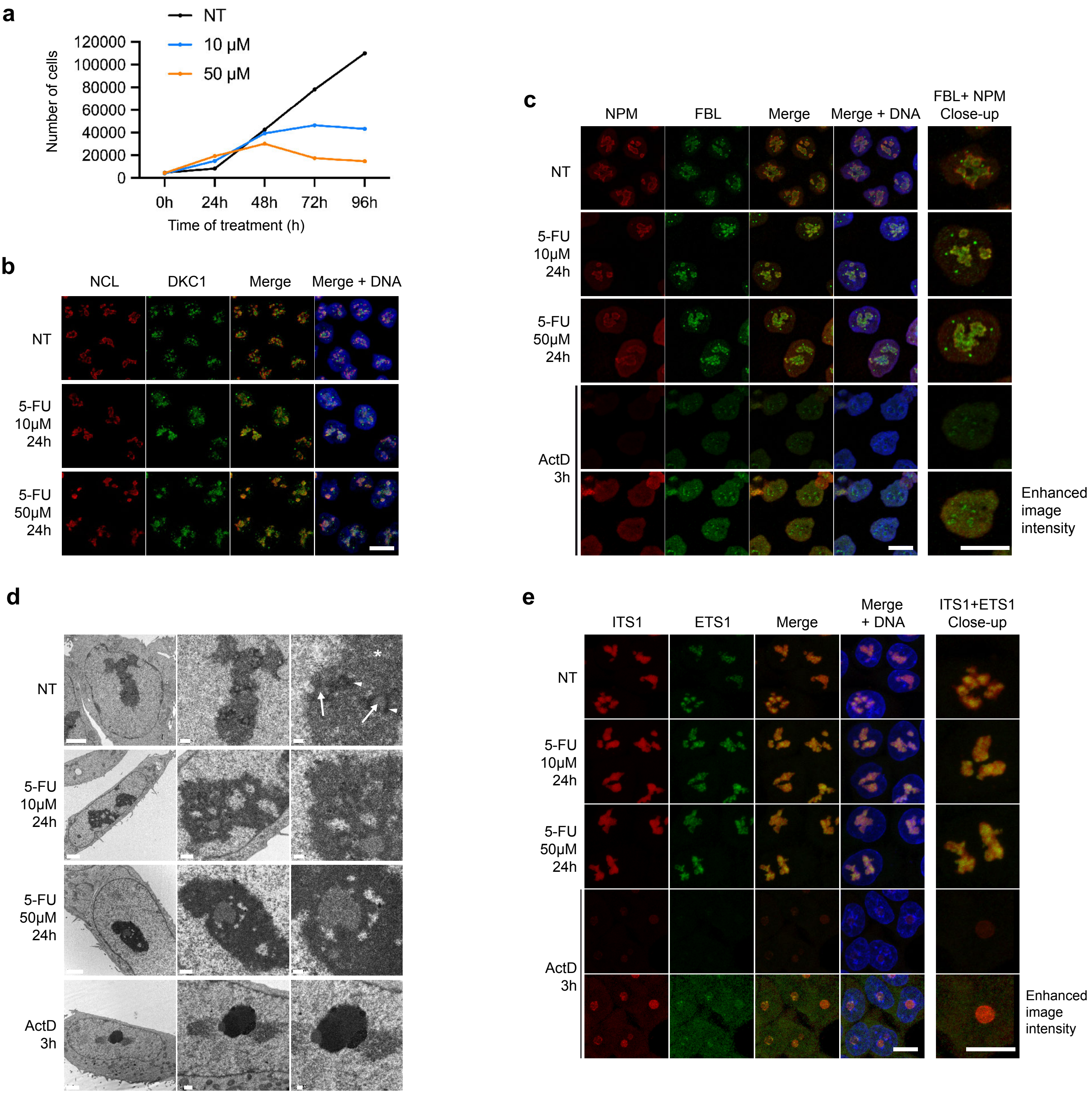

Supplementary Figure 1, related to Figure 1: Ribosome production is maintained in 5-FU treated cells.
a, HCT116 were either untreated (NT, black) or treated with 10 µM (light blue) or 50 µM (orange) of 5-FU for the indicated time. Cells were stained with Hoechst and nuclei were counted by high content analysis imaging. Data are number of cells per well. **b-e,** HCT116 cells were either untreated (NT) or treated with 10 µM 5-FU for 24 h, or with 50 ng/mL actinomycin D for 3 h (ActD). **b,c,** Confocal microscopy imaging following immunofluorescence detection of nucleolin (NCL) fibrillarin (FBL), nucleophosmin (NPM) and dyskerin (DKC1). DNA was stained with Hoechst (blue). The right column shows a close-up of one nucleus. To illustrate the dispersal of NPM upon actinomycin D treatment the signal was enhanced (bottom row). The images show that under 5-FU, nucleoli are enlarged, FBL and DKC1 did not reorganise as nucleolar cap and NCL and NPM did not disperse in the nucleoplasm, as it is the case when pre-rRNA synthesis is inhibited (see ActD as a control). These observations are consistent with a maintenance of pre-rRNA synthesis and alteration of processing. Scale bar = 10 µM. **d**, Nucleolus ultra-structure imaged by transmission electron microscopy. Arrows = Fibrillar center; arrow heads = Dense fibrillar component; star = Granular component. Scale: left column = 2 µm, middle column = 0.5 µm, right column = 0.2 µm. TEM data shows that nucleoli ultra-structure is modified and reflects an alteration of ribosome maturation, and not an inhibition of pre-rRNA synthesis. **e,** Confocal microscopy imaging following fluorescent in situ hybridization (FISH) with probes detecting only the 5'-ETS and ITS1 regions of the pre-rRNAs (see Extended data Fig. 2a). DNA was stained with Hoechst (blue). The right column shows a close-up of one nucleus. The ITS1 probe detect several pre-rRNA species and serves as a reference. The 5'ETS probe detect only the 47S and 45S pre-rRNAs and reflect rRNA synthesis. Inhibition of pre-rRNA synthesis by actinomycin D induce a loss of signal with both 5'-ETS and ITS1 probes. In contrast, upon 5-FU treatment, the signal from both probes was detected at level similar to the untreated control cells. These observations are consistent with a maintenance of pre-rRNA synthesis. Scale bar = 10 µM.

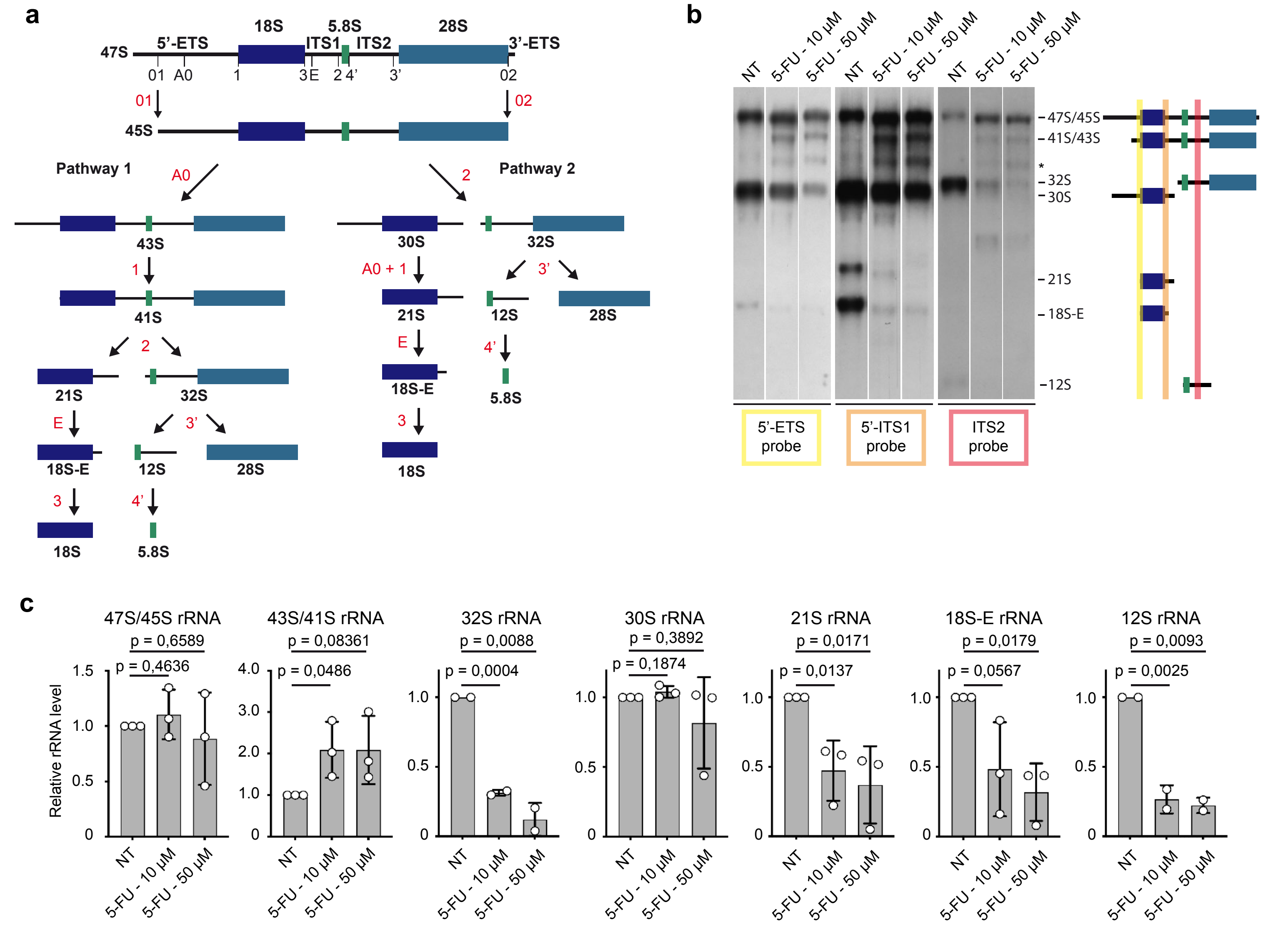

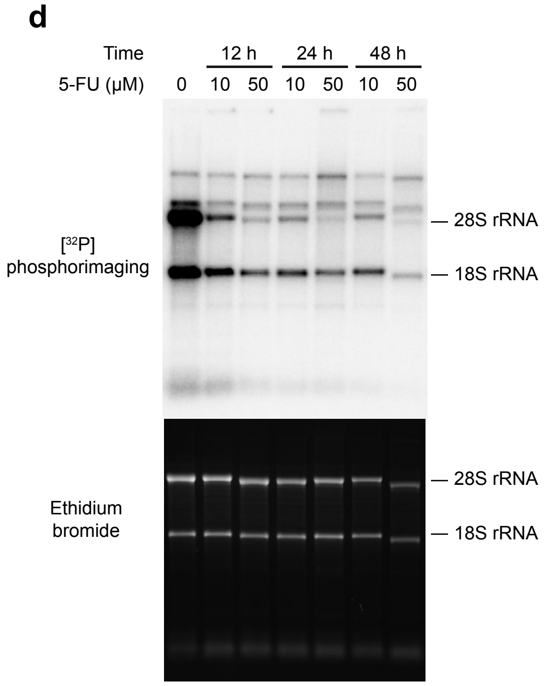

Supplementary Figure 2, related to Figure 1: Ribosome production is maintained in 5-FU treated cells.

a, Simplified representation of pre-rRNA processing adapted from 1. Cleavage sites are indicated in black on the full length 47S pre-rRNA. The products of each cleavage site (in red) are indicated.

b, Northern-blot analysis on whole cell RNA from HCT116 cells using probes detecting 5'-ETS, ITS1 or ITS2 spacer regions. The position of the probes and the detected pre-rRNA intermediates are indicated on the right. The star indicates a pre-rRNA intermediate not yet identified.

c, Quantification of rRNA intermediates detected by northern-blot, from independent experiments. Signals were normalized against the intensity of 18S rRNA band detected by ethidium bromide. Data are expressed as mean +/- s.d, and individual data are shown. n = 3, except for 32S and 12S, n = 2. Results of unpaired two-tailed t-test are indicated. These data show that the 47S/45S precursors are produced at similar level in 5-FU treated cells compared to non-treated cells. Increase of 43S/41S intermediates and decrease in 32S and 21S intermediates reflect a processing defect at cleavage site 2.

d, Representative images of [^{32}P] pulse-chase analysis presented in Fig. 1C, which was performed three times. Position of 18S and 28S rRNAs are indicted on the right. 28S and 18S rRNA bands in ethidium bromide gel were used as markers (indicated on the left).

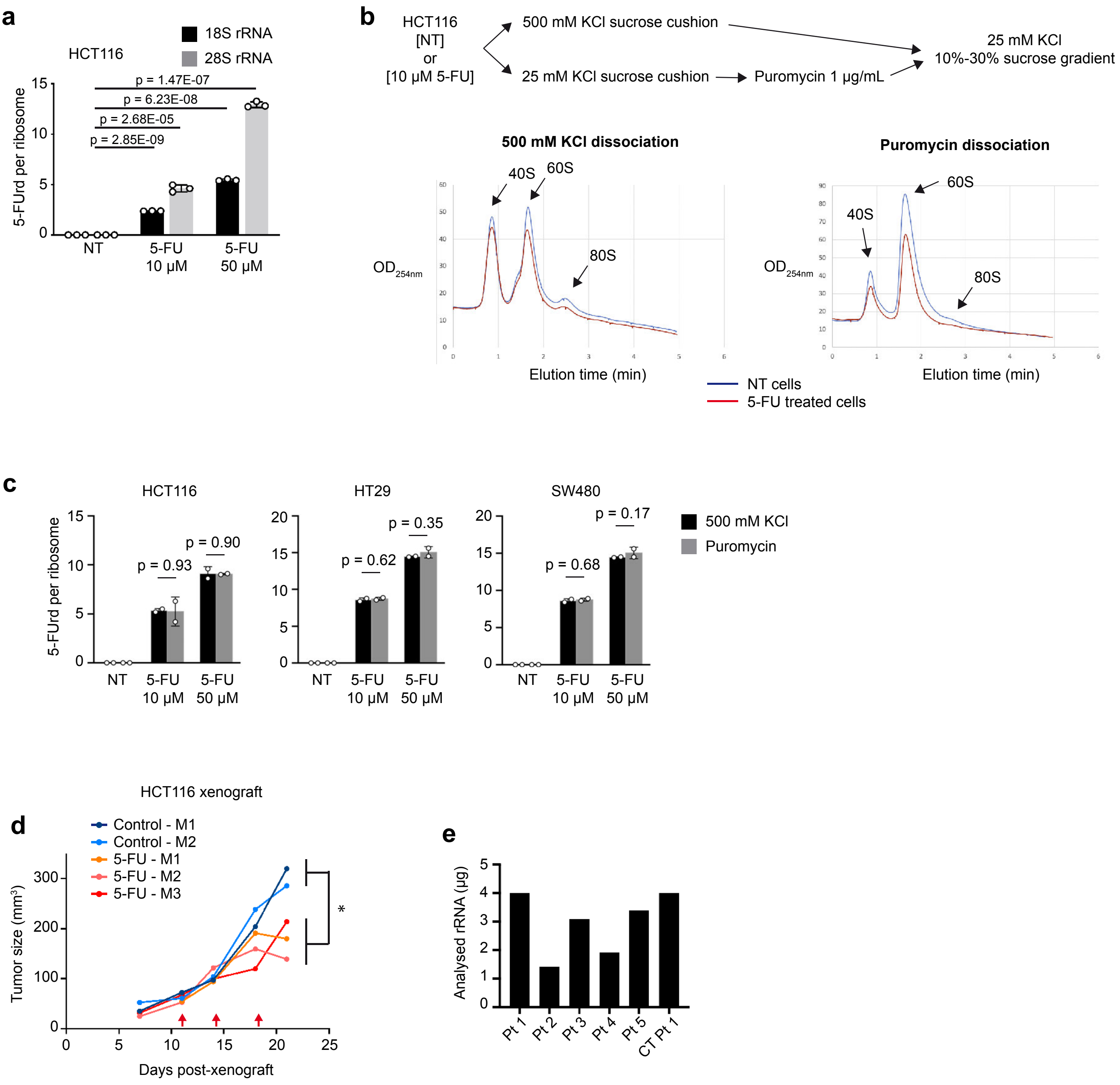

Supplementary Figure 3, related to Figure 2: incorporation of 5-FU in cell lines, xenografts and human tumours.

a, Quantification of 5-FUrd in rRNA by LC-HRMS from 18S and 28S rRNAs that were gel-purified. This approach provides a level of 5-FUrd incorporation similar to that of rRNA extracted from purified ribosomes. Data are from 3 independent experiments. Data are expressed as mean +/- s.d. n = 3 and individual points are shown. Results of unpaired two-tailed t-test are indicated.

b, HCT116 Ribosomal subunit profiles from sucrose gradient after dissociation either on a 500 mM KCl sucrose cushion, either by puromycin treatment. The scheme on top summarize the experimental setting. The peaks of the individual subunit and of the 80S ribosome are indicated. The data show that a small 80S peak is present in the profile of 500 mM KCl purified ribosomes, and is undetected in the profile of ribosomes dissociated with puromycin.

c, Quantification of 5-FUrd in rRNA by LC-HRMS from ribosomal subunit purified either directly on a 500 mM KCl sucrose cushion, either by puromycin treatment followed by a 500 mM KCl sucrose cushion. The data show the same level of 5-FUrd for both method. The experiment was performed on HCT116, HT29 and SW480 to exclude any cell line effect. Data are expressed as mean +/- s.d. n = 2. Individual points are shown.

d, HCT116 Xenograft growth curves for individual tumours. Tumor size was measured weekly and are shown in mm^3. The results showed that in response to 5-FU treatment, tumour growth is slowed-down. Arrows indicate 5-FU treatments. Result of unpaired two-tailed t-test is indicated. Individual points are shown.

e, Analysis of human colorectal tumour samples. Quantity of rRNA (µg) used in LC-HRMS analysis. For all samples, the quantity of analysed RNA was higher than for our standard protocol (1 µg or rRNA).

a HCT116 cells

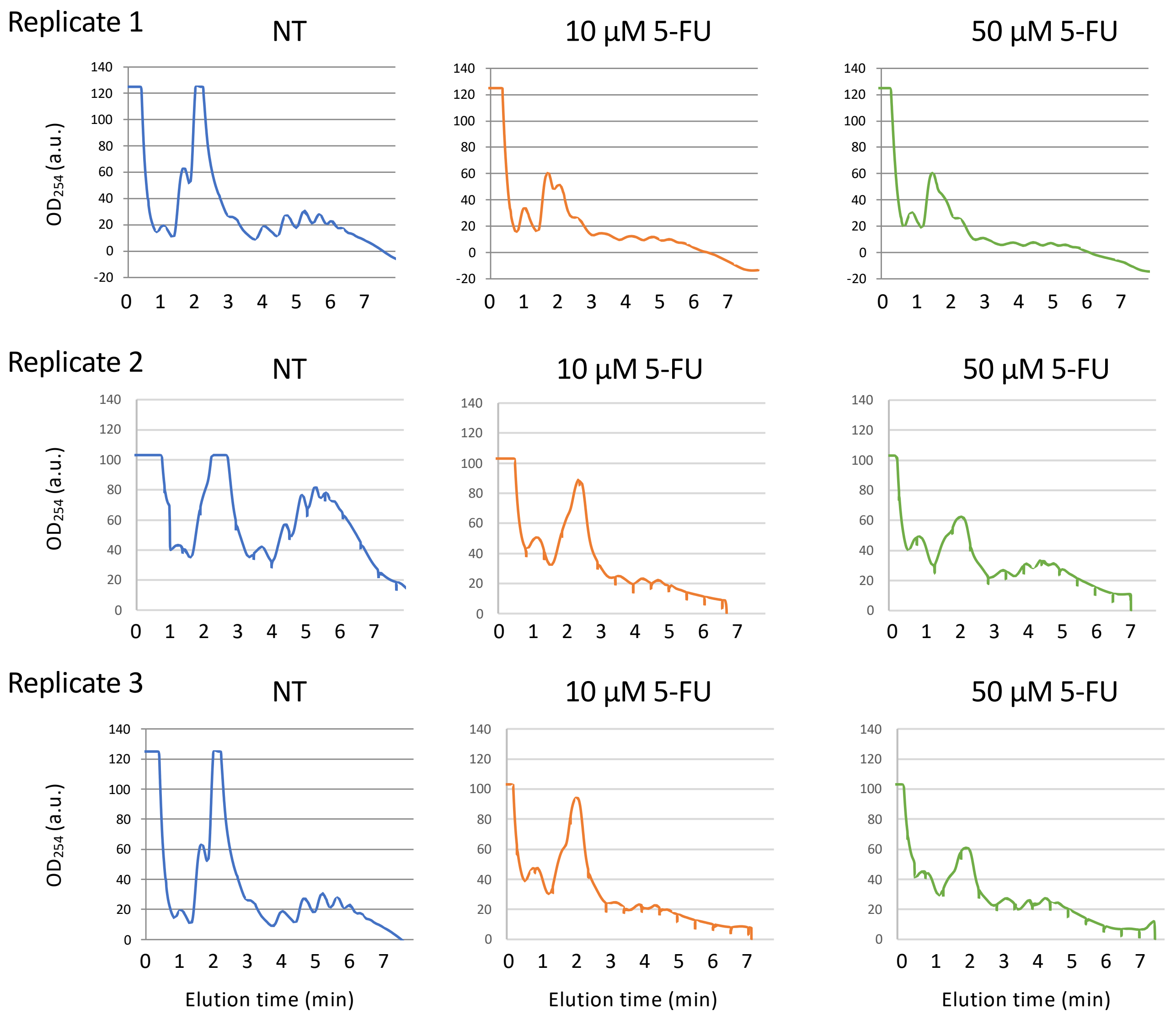

b HT29 cells

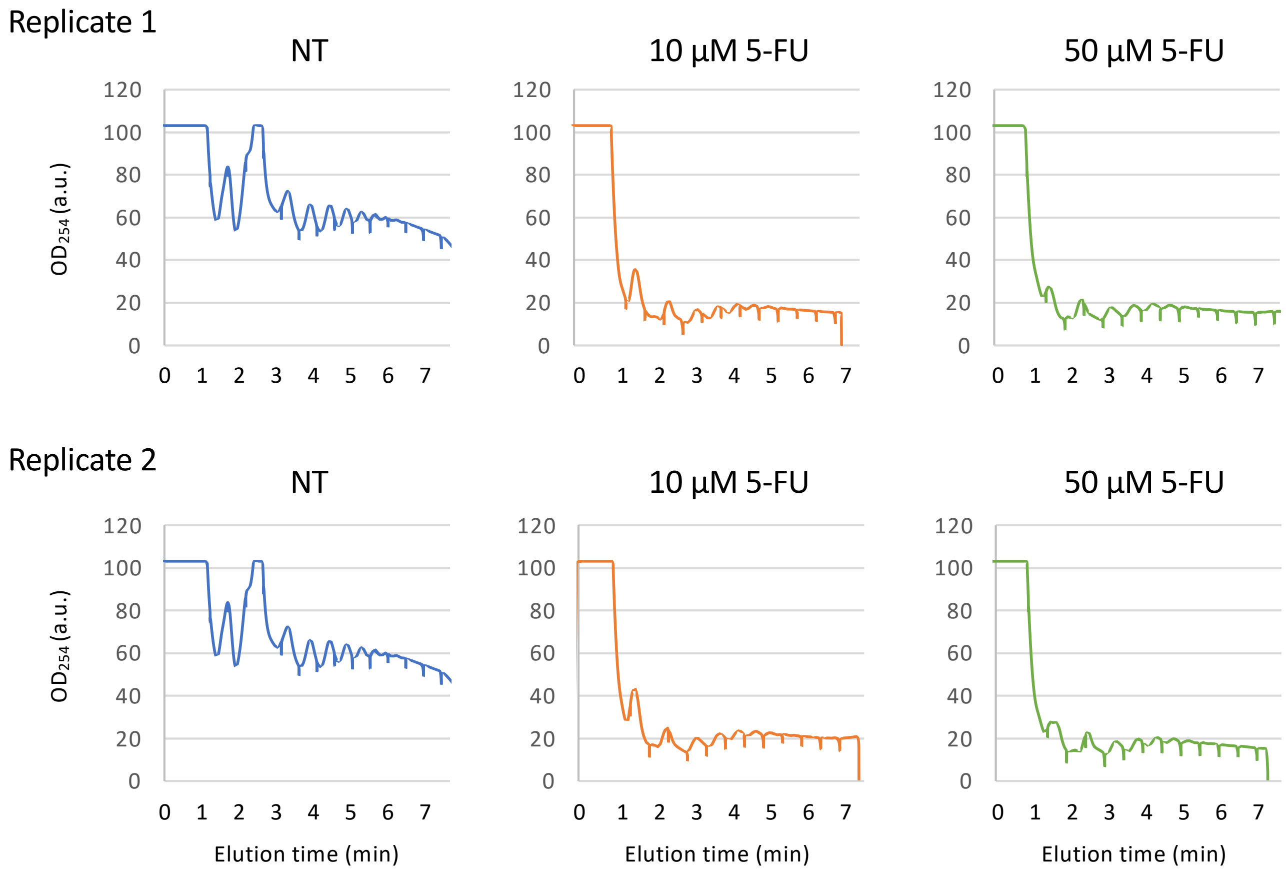

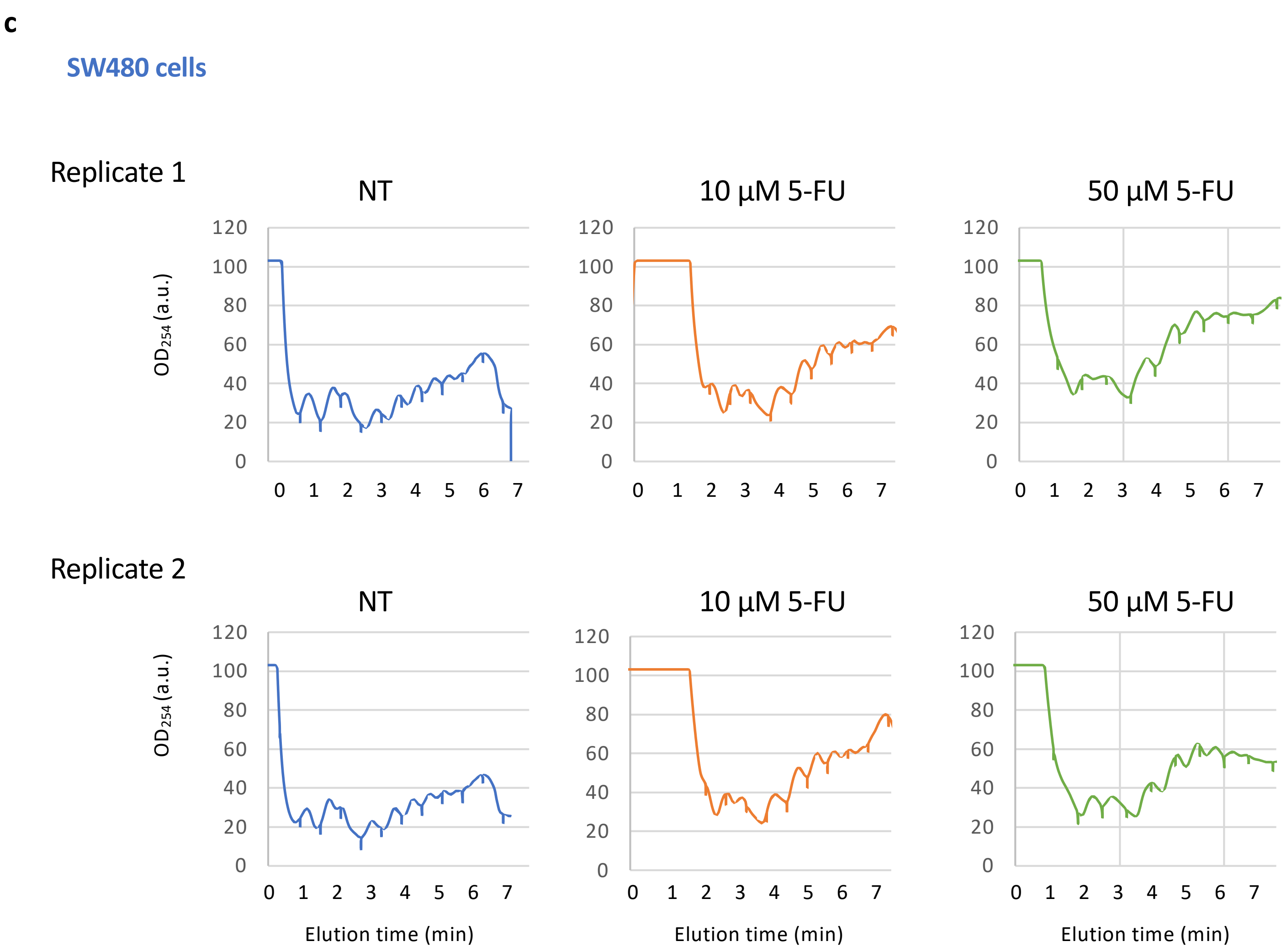

Supplementary Figure 4 - Polysome profiles related to Figure 3a

Profile of cytoplasmic ribosome sucrose gradient fractionation of each biological replicate used in Figure 3a. **a,** HCT116 cells ; **b,** HT29 cells ; **c,** SW480 cells

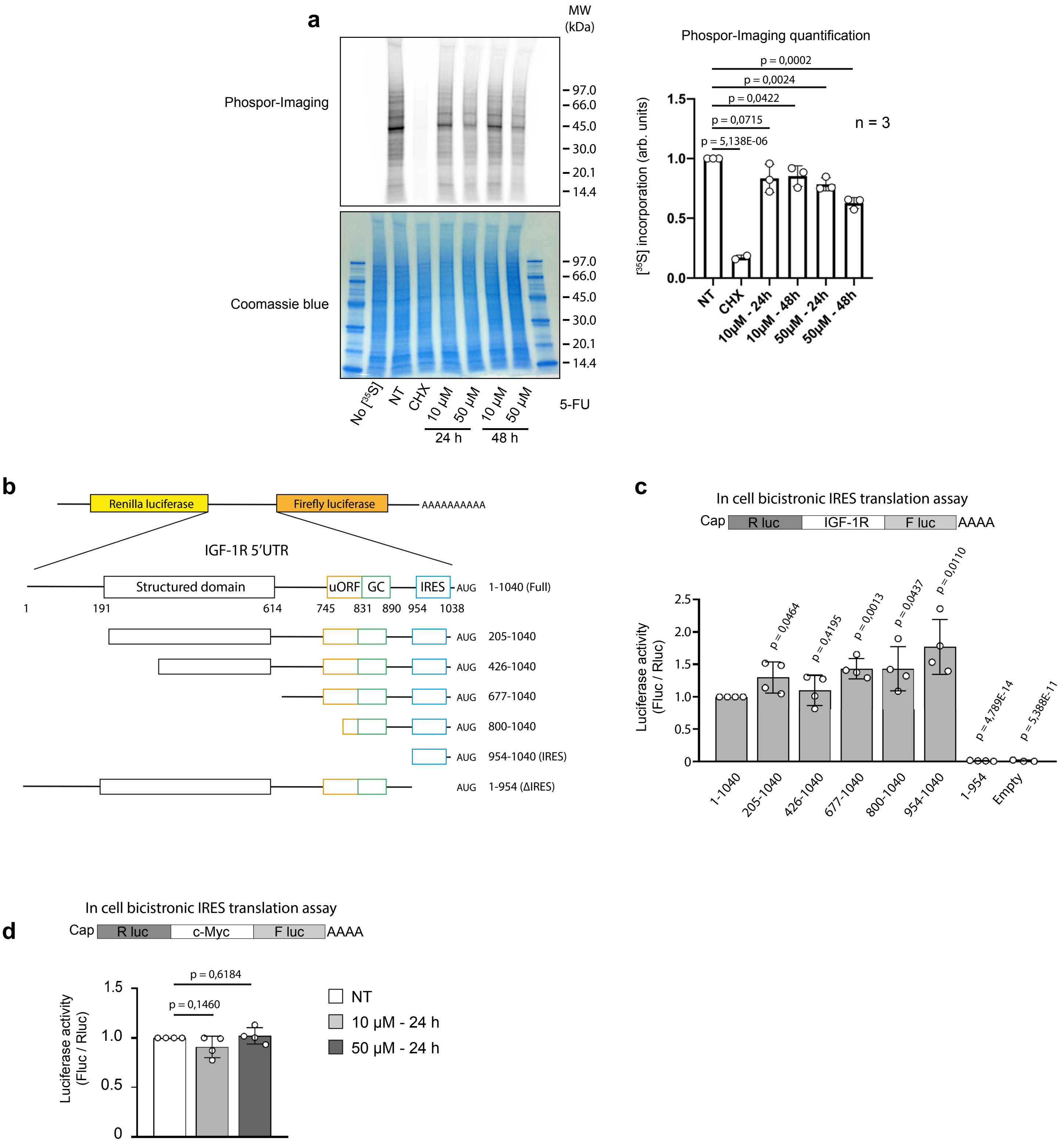

Supplementary figure 5, related to Figure 3. Altered translation by Fluorinated ribosomes.

a, Measure of global protein synthesis by [35S]-Met-Cys pulse labelling. HCT116 cells were treated with 10 µM or 50 µM 5-FU for 24 h or 48 h or untreated (NT) or treated with protein synthesis inhibitor cycloheximide at 50 µg/mL for 30 min (CHX). Cells were pulse-labelled with [35S]-Met-Cys) for 30 min before cell harvesting, except in the negative control (No [35S]). 10 µg of total protein was analysed by SDS-PAGE (left), and the SDS-PAGE gel was dried and exposed to phosphor-imaging screen (top left). The radioactivity signal was quantified from phosphor-imaging, and was plotted (right). Each dot represents an individual biological sample measured in duplicate and data are expressed as mean ± s.d, and individual measure are indicated. Results of unpaired two-tailed t-test are indicated on top of the pannel.

b, Schematic representation of the human IGF-1R 5'UTR and of the deletion constructs used inserted in the bicistronic reporter vactor (shown on top) and used in panel **c**. **c**, HCT116 cells were transfected with bicistronic reporter vectors containing the IGF-1R construct represented in **b**. The IRES activity is represented as the LucF / LucR ratio, normalized to the activity of full length IGF-1R 5'UTR. Data are mean values ± s.d. of 4 independent biological replicates (each performed in triplicate). Individual data points represented. Results of unpaired two-tailed t-test for each deletion compared to the full length 5'UTR are indicated on top of the pannel. **d**, Same experiment as in Fig. 3e, using a reporter vector monitoring c-Myc 5'UTR IRES activity. Results of unpaired two-tailed t-test are indicated.

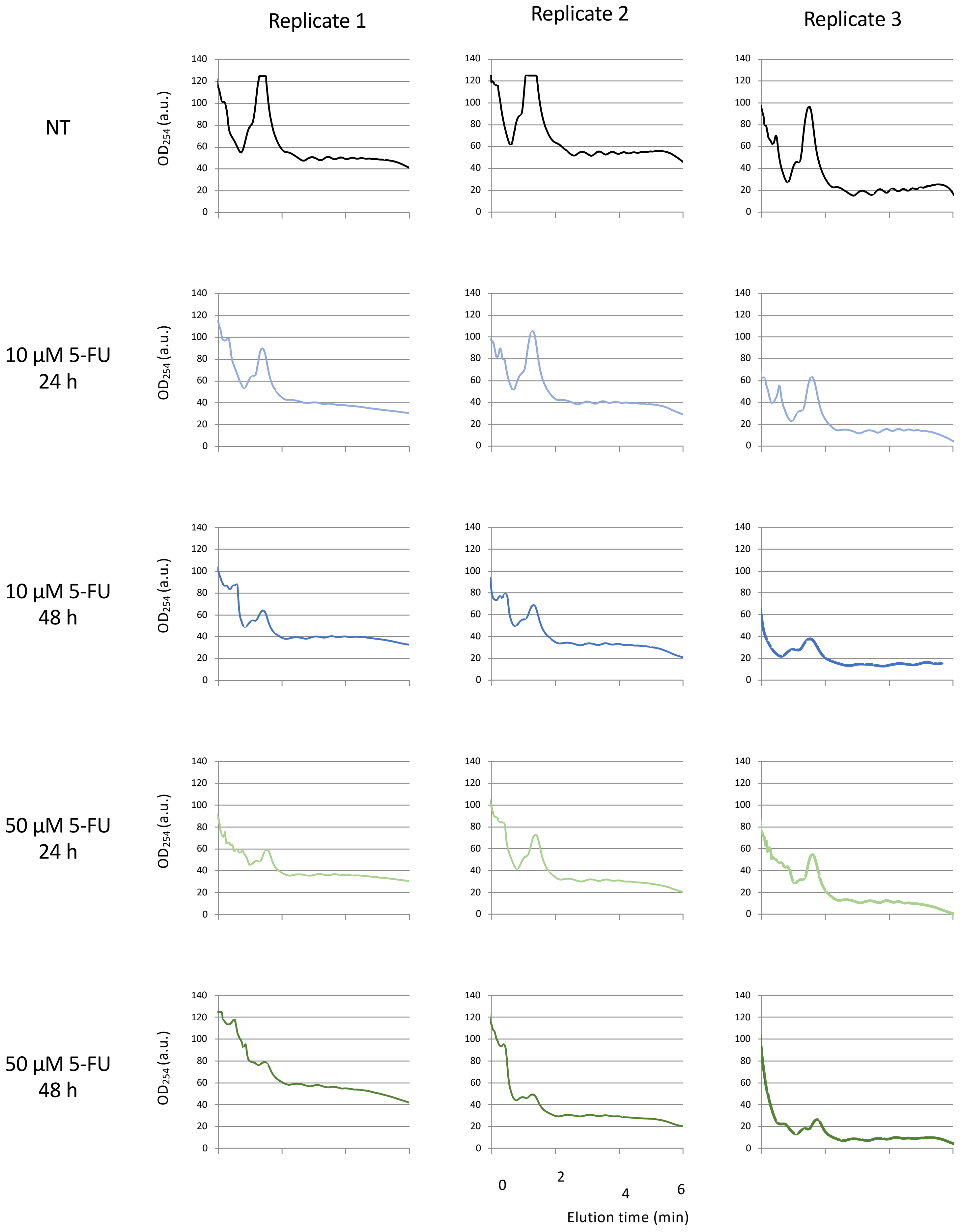

Supplementary Figure 6 - Polysome profiles related to Figure 4a
Profile of cytoplasmic ribosome's sucrose gradient fractionation of individual biological replicate used in Figure 4a.

a

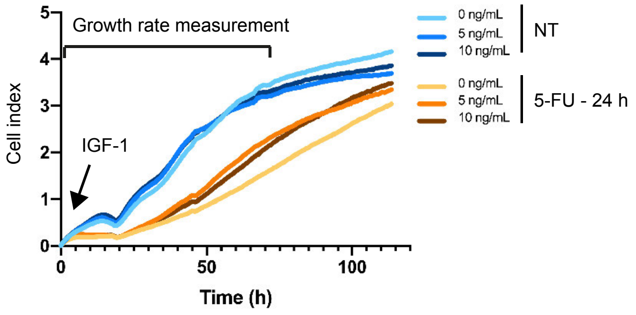

Supplementary Figure 7, related to Figure 4. IGF-1R contributes to survival and recovery of 5-FU treated CRC cells.
a, Same experiment as Fig. 4d and e. Shown are individual cell growth curves for each condition. The period used to calculate the growth rate (Fig. 4d) is indicated on top. Each curve is the mean of a technical triplicate (No IGF-1, n = 2 ; IGF-1 treated, n = 3).

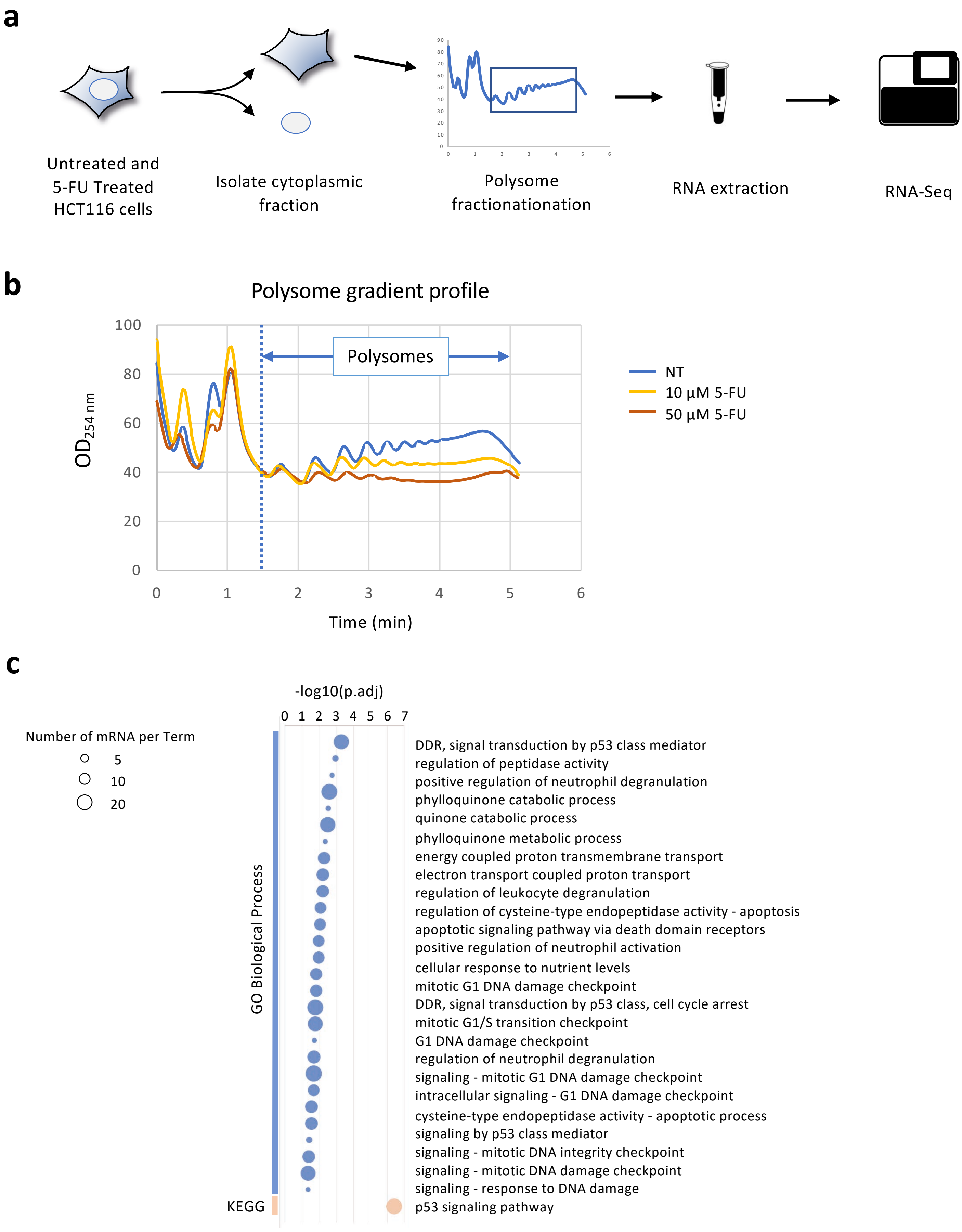

Supplementary Figure 8, related to Figure 5. Polysome profiling of HCT116 cells treated with 5-FU.

a. Overview of the polysome profiling experiment.

b. Typical polysome profile of HCT116 either untreated or treated with 5-FU at 10 µM or 50 µM for 24 h. Complete profile dataset is avaiblable in Supplementary data 5. The fractions corresponding to "polysomal" are indicated.

c. GO term analysis of all translationally altered transcripts in HCT116 cells upon 10 µM 5-FU treatment for 24h. Dot surface reflects the intersection size.

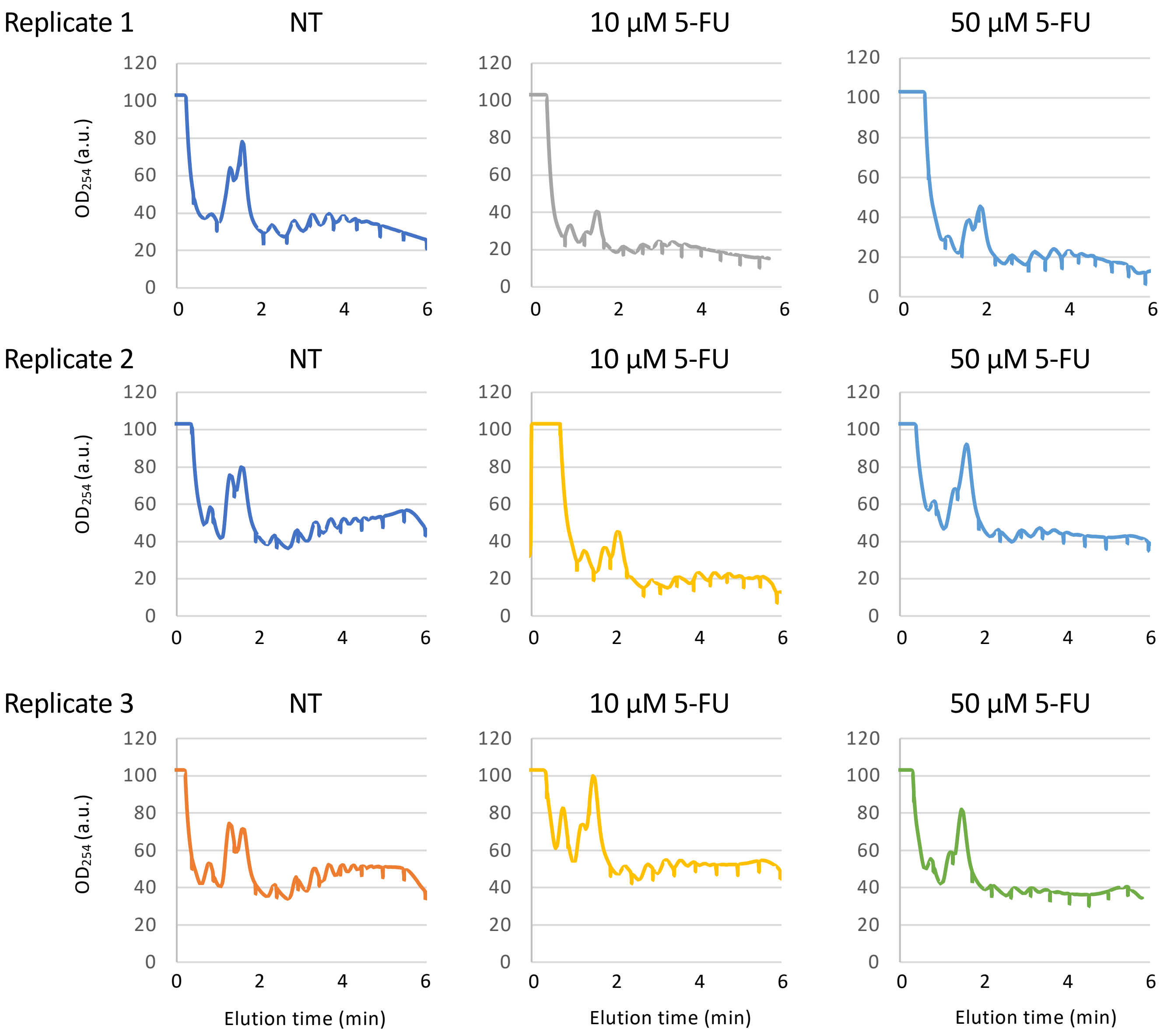

Supplementary Figure 9 - Polysome profiles related to Figure 5a and 5b

Profile of cytoplasmic ribosome sucrose gradient fractionation of each biological replicate used in Figure 5a and 5b.
