# Supplementary material for: Alteration of ribosome function upon 5-fluorouracil treatment favors cancer cell drug-tolerance
Source: Nat Commun. 2022 Jan 10;13:173. doi: 10.1038/s41467-021-27847-8 (PMC8748862; doi:10.1038/s41467-021-27847-8)
Supplement: Supplementary file 3 — Description of Additional Supplementary Files [file 41467_2021_27847_MOESM3_ESM.pdf]

Description of Additional Supplementary Files

File Name: Supplementary Data 1
Description: Related to Figure 5a, 5b and 5c. Translatome data used to prepare Figure 5a (10µM 5-FU vs NT) and Figure 5b (50 µM 5-FU vs NT). The table contains for each mRNA the Essembl identifier, the Log2 fold-change of mRNA abundance in 5-FU treated vs untreated cells polysomal fraction, the adjusted *p* value, and the gene symbol. See legend of Figure 5a-5b for details.

File Name: Supplementary Data 2
Description: Related to Figure 5d. Gene Ontology (GO) terms used to prepare figure 5d. The table contains the GO terms for biological process, and the KEGG term, their GO ID number, the associated adjusted *p* value and number of mRNA for each term. See legend of Figure 5d for details.

File Name: Supplementary Data 3
Description: Related to Figure 5e and 5f. GO Term classes used to prepare Figure 5e and 5f. The table contains the significantly enriched GO class, their ID number, the number of terms per class and the corresponding fraction (%) compared to the total number of GO terms. See legend of Figure 5d for details.

File Name: Supplementary Data 4
Description: Related to Figure 5. List of survival gene mRNAs identified within the groups of mRNA significantly increased or decreased in the polysomal fractions of HCT116 cells treated with 10 µM or 50 µM of 5-FU compared to untreated cells (available in Supplementary Data 1).

File Name: Supplementary Data 5
Description: Related to Figure 5. Result of functional annotation clustering of up-translated survival genes mRNA (see Supplementary Data 4) using the DAVID tool. The table contains the GO term of each cluster, the number of term per cluster and the *P* value.
