# Supplementary material for: Alteration of ribosome function upon 5-fluorouracil treatment favors cancer cell drug-tolerance
Source: Nat Commun. 2022 Jan 10;13:173. doi: 10.1038/s41467-021-27847-8 (PMC8748862; doi:10.1038/s41467-021-27847-8)
Supplement: Supplementary file 9 — Reporting Summary [file 41467_2021_27847_MOESM9_ESM.pdf]

nature portfolio

Corresponding author(s): Jean-Jacques DIAZ & Frédéric CATEZ

Last updated by author(s): Nov 23, 2021

Reporting Summary

Nature Portfolio wishes to improve the reproducibility of the work that we publish. This form provides structure for consistency and transparency in reporting. For further information on Nature Portfolio policies, see our Editorial Policies and the Editorial Policy Checklist.

Statistics

For all statistical analyses, confirm that the following items are present in the figure legend, table legend, main text, or Methods section.

n/a	Confirmed	
☐	☒	The exact sample size (n) for each experimental group/condition, given as a discrete number and unit of measurement
☐	☒	A statement on whether measurements were taken from distinct samples or whether the same sample was measured repeatedly
☐	☒	The statistical test(s) used AND whether they are one- or two-sided<br>*Only common tests should be described solely by name; describe more complex techniques in the Methods section.*
☒	☐	A description of all covariates tested
☐	☒	A description of any assumptions or corrections, such as tests of normality and adjustment for multiple comparisons
☐	☒	A full description of the statistical parameters including central tendency (e.g. means) or other basic estimates (e.g. regression coefficient) AND variation (e.g. standard deviation) or associated estimates of uncertainty (e.g. confidence intervals)
☐	☒	For null hypothesis testing, the test statistic (e.g. F, t, r) with confidence intervals, effect sizes, degrees of freedom and P value noted<br>*Give P values as exact values whenever suitable.*
☒	☐	For Bayesian analysis, information on the choice of priors and Markov chain Monte Carlo settings
☒	☐	For hierarchical and complex designs, identification of the appropriate level for tests and full reporting of outcomes
☒	☐	Estimates of effect sizes (e.g. Cohen's d, Pearson's r), indicating how they were calculated

Our web collection on statistics for biologists contains articles on many of the points above.

Software and code

Policy information about availability of computer code

Data collection	For immunofluorescence: ZEN black software (Carl Zeiss). For phosphorImaging: ImageQuant TL (GE HealthCare). For High Content analysis: Harmony software (Perkin Elmer). For NGS, NextSeq System Suite v2.2.0 (Illumina). For western blot : ImageLab 6.0 (BioRad)
Data analysis	For immunofluorescence and quantification of northern blot: ImageJ (build bad6864e55 - https://imagej.net). For High Content Analysis: Columbus software (Perkin Elmer). Calculations were performed with Microsoft Excel (version 16.15). Statistical analyses were performed with PRISM (Version 7.0 - GraphPad). For NGS analysis, FastQC v0.11.9 (Babraham Institute, Cambridge, UK), Cutadapt v3.2, STAR v2.7.7, HTSeq-count v0.13.5, Wald test from DESeq2 R package, gProfiler on Ensembl release v103, CateGOrizer v3.218, DAVID v 6.8.

For manuscripts utilizing custom algorithms or software that are central to the research but not yet described in published literature, software must be made available to editors and reviewers. We strongly encourage code deposition in a community repository (e.g. GitHub). See the Nature Portfolio guidelines for submitting code & software for further information.

Data

Policy information about availability of data

All manuscripts must include a data availability statement. This statement should provide the following information, where applicable:

- Accession codes, unique identifiers, or web links for publicly available datasets
- A description of any restrictions on data availability
- For clinical datasets or third party data, please ensure that the statement adheres to our policy

RNA sequencing data of translatome analysis generated in this study are available at the NCI Gene Expression Omnibus database under accession number GSE178839. Datasets of translatome analysis and polysome profiles are available within supplementary data. All data used to generate the figures and tables are

provided as supplementary data and in the "Source data" file accompanying this paper. Any other data supporting the findings and biological materials are available from the corresponding author upon reasonable request.

Field-specific reporting

Please select the one below that is the best fit for your research. If you are not sure, read the appropriate sections before making your selection.

☒ Life sciences ☐ Behavioural & social sciences ☐ Ecological, evolutionary & environmental sciences

For a reference copy of the document with all sections, see nature.com/documents/nr-reporting-summary-flat.pdf

Life sciences study design

All studies must disclose on these points even when the disclosure is negative.

Sample size	To obtain data for statistical analysis, most of the experiments included 2 or 3 technical replicates and were repeated in at least three independent experiments. Analysis on animal and human samples were performed once and are reported individualy.
Data exclusions	No data were excluded
Replication	Most of the experiments were performed at least three times unless otherwise stated. Statistical analysis was performed to verify significance. All attempts of replication were successful. The number of replication is indicated in each figure and/or legend.
Randomization	Age- and gender-matched mice were randomly allocated to different experimental groups.
Blinding	We report in vitro analyses where blinding is not required. However, data analysis of 5-FU incorporation was performed independently by 3 investigators.

Reporting for specific materials, systems and methods

We require information from authors about some types of materials, experimental systems and methods used in many studies. Here, indicate whether each material, system or method listed is relevant to your study. If you are not sure if a list item applies to your research, read the appropriate section before selecting a response.

Materials & experimental systems

n/a	Involved in the study	
☐	☒	Antibodies
☐	☒	Eukaryotic cell lines
☒	☐	Palaeontology and archaeology
☐	☒	Animals and other organisms
☐	☒	Human research participants
☒	☐	Clinical data
☒	☐	Dual use research of concern

Methods

n/a	Involved in the study	
☒	☐	ChIP-seq
☒	☐	Flow cytometry
☒	☐	MRI-based neuroimaging

Antibodies

Antibodies used	Antibodies used for immuno-fluorescence: anti-FBL rabbit polyclonal antibody (ab5821, Abcam) diluted at 1:2,000, anti-DKC1 rabbit polyclonal (sc-48794, Santa Cruz Biotechnology) diluted at 1:500 and anti-NCL mouse monoclonal antibody [4E2] (ab13541, Abcam) at 1:4,000. Secondary antibodies were used at 1:1,000: Goat anti-Mouse IgG (H+L) Cross-Adsorbed Secondary Antibody, Alexa Fluor 488 (A-11001, Thermofisher Scientific), Goat anti-Rabbit IgG (H+L) Cross-Adsorbed Secondary Antibody, Alexa Fluor 488 (A-11008, Thermofisher Scientific), Goat anti-Mouse IgG (H+L) Cross-Adsorbed Secondary Antibody, Alexa Fluor 555 (A-21422, Thermofisher Scientific), and Goat anti-Rabbit IgG (H+L) Cross-Adsorbed Secondary Antibody, Alexa Fluor 555 (A-21428, Thermofisher Scientific). For western blotting: anti-GAPDH mouse monoclonal Antibody [6C5] (AM4300, Invitrogen) diluted at 1:5000 and rabbit monoclonal antibodies against IGF-1R (#9750, Cell Signalling) diluted at 1:1000 anti-Histone H3 (ab1791, Abcam) diluted at 1:2000 and anti-actin (ab179467, Abcam) diluted at 1:2000. Western, blot secondary antibodies were Horseradish peroxidase conjugated secondary antibodies Anti-rabbit IgG, HRP-linked Antibody (#7074, Cell Signalling Technologies) and Anti-mouse IgG, HRP-linked Antibody (#7076, Cell Signalling Technologies) diluted at 1:5000.
Validation	The following antibodies were validated according to manufacturer's manual : anti-FBL rabbit polyclonal antibody (ab5821, Abcam), anti-DKC1 rabbit polyclonal (sc-48794, Santa Cruz Biotechnology), anti-NCL mouse monoclonal antibody (ab13541, Abcam), anti-GAPDH (AM4300, Invitrogen) anti-IGF-1R (#9750, Cell Signalling) and anti-actin (ab179467, Abcam), and secondary antibodies were labelled with AlexaFluor 488 (A-11001 and A-11008) or AlexaFluor 555 (A-21422 and A-21428) (Molecular Probes).<br>All antibody solution were validated in the laboratory within this manuscript and in our previous publications Marcel, V. et al. Cancer Cell, 24, 318–330 (2013), and Erales, J. et al. Proc. Natl. Acad. Sci. 114, 12934–12939 (2017).

Eukaryotic cell lines

Policy information about cell lines

Cell line source(s)	The following cell lines were obtained from ATCC: HCT116 (ATCC CCL-247), MDA-MB-231 (ATCC HTB-26), BT20 (ATCC HTB-19), HT29 (ATCC HTB-38), SW480 (ATCC CCL-228), Panc1 (ATCC CRL-1469) and MiaPaCa (ATCC CRL-1420). The following cell lines were obtained from the authors at ISRECO institute: ISRECO1/2/3 (Cajot, J. F., et al. Cancer Res. (1997) 57, 2593–2597)
Authentication	The following cell lines were authenticated by 21 PCR-single-locus-technology (Eurofins, Ebersberg, Germany): HCT116, HT29, MDA-MB-231, MiaPaCa, Panc1. ISRECO1 cell line was not authenticated by PCR-single-locus-technology as this cell line genetic pattern is not described in databases.
Mycoplasma contamination	All the cell lines were routinely tested negative against mycoplasma infection using MycoAlert kit (Promega)
Commonly misidentified lines (See ICLAC register)	No commonly misidentified cell lines were used.

Animals and other organisms

Policy information about studies involving animals; ARRIVE guidelines recommended for reporting animal research

Laboratory animals	Experiments were performed with 7 weeks old female Hsd: Athymic Nude-Foxn1nu mice (Envigo). Each animal was 20 g-25 g of weight. Mice were housed with access to water and food ad libitum, with a light / dark cycle of 12h / 12h. Housing was at about 23°C and 50% humidity.
Wild animals	The study did not involve wild animals
Field-collected samples	The study did not involve field-collected samples
Ethics oversight	In vivo experiments followed French guidelines for experimental animal studies (DSV agreement A34-172-13).

Note that full information on the approval of the study protocol must also be provided in the manuscript.

Human research participants

Policy information about studies involving human research participants

Population characteristics	Samples were selected based on whether the patient received a 5-FU based chemotherapy before surgery and tumor resection. The delay between chemotherapy and surgery varied from 1.5 to 3.5 months. As an opportunistic collection of data age, gender and genetic information were not used as selection criteria. This information is not part of the current manucript, and can be added during the revision process.
Recruitment	Recruitment and tumor samples collection was performed in accordance with French regulation and following standard operating procedures. All patients signed an informed consent.
Ethics oversight	Human samples were used under clinical agreement #NCT01577511 under authority of Nîmes Carrémeau University Hospital.

Note that full information on the approval of the study protocol must also be provided in the manuscript.
